# Comprehensive Experimental System for a Promising Model Organism Candidate for Marine Teleosts

**DOI:** 10.1038/s41598-019-41468-8

**Published:** 2019-03-20

**Authors:** Keishi Sakaguchi, Michio Yoneda, Noriyoshi Sakai, Kanako Nakashima, Hajime Kitano, Michiya Matsuyama

**Affiliations:** 1 Fisheries Research Institute of Karatsu, Department of Joint Research, Faculty of Agriculture, Kyushu University, Saga, 847-0132 Japan; 2 National Research Institute of Fisheries and Environment of Inland Sea, Hakatajima Station, Imabari, 794-2305 Japan; 3 Genetic Strains Research Center, National Institute of Genetics, Mishima, 411-8540 Japan; 4 Department of Genetics, School of Life Sciences, SOKENDAI (The Graduate University for Advanced Studies), Mishima, 411-8540 Japan; 5 Laboratory of Marine Biology, Department of Bioresource Sciences, Faculty of Agriculture, Kyushu University, Fukuoka, 819-0385 Japan

## Abstract

A comprehensive experimental system for Japanese anchovy, a promising candidate model organism for marine teleosts, was established. Through the design of a rearing/spawning facility that controls the photoperiod and water temperature, one-cell eggs were continuously obtained shortly after spawning throughout the rearing period. The stages of eggs are indispensable for microinjection experiments, and we developed an efficient and robust microinjection system for the Japanese anchovy. Embryos injected with *GFP* mRNA showed strong whole-body GFP fluorescence and the survival rates of injected- and non-injected embryos were not significantly different, 87.5% (28 in 32 embryos) and 90.0% (45 in 50 embryos), respectively. We verified that the *Tol2* transposon system, which mediates gene transfer in vertebrates, worked efficiently in the Japanese anchovy using the transient transgenesis protocol, with *GFP* or DsRed as the reporter gene. Finally, we confirmed that genome-editing technologies, namely Transcription Activator-Like Effector Nucleases (TALEN) and Clustered Regulatory Interspaced Short Palindromic Repeats (CRISPR)/Cas9, were applicable to the Japanese anchovy. In practice, specific gene-disrupted fishes were generated in the F_1_ generation. These results demonstrated the establishment of a basic, yet comprehensive, experimental system, which could be employed to undertake experiments using the Japanese anchovy as a model organism for marine teleost fish.

## Introduction

A model organism is a non-human species that is extensively studied to understand specific biological phenomena, with the expectation that investigations made in the model organism will provide insights into the mechanisms operating in other organisms that are difficult to study directly^1^. Studies utilising model organisms are an efficient and effective approach because such organisms have several experimental advantages in a laboratory setting, such as a manageable size, ease of maintenance and breeding, straightforward observation and a short generation time. Hence, a diverse range of model organisms has been established ranging from viruses to mammals and plants. Researchers can select the most appropriate model organism and develop their research based upon the abundant biological data and sophisticated experimental methods established by previous studies. However, it is often difficult to use existing models for the analysis of a large variety of biological phenomena; therefore, occasionally it becomes necessary to conduct research using non-model organisms.

Among teleosts, small freshwater fish species, zebrafish (*Danio rerio*) and medaka (*Oryzias latipes*), are the most utilised model organisms. However, when referencing previous studies on teleosts, particularly marine teleosts, including many species important for the aquaculture and the fisheries industry, research is confined to the targeted fish species itself. There could be several reasons for this as follows: (1) there are no appropriate model organisms; (2) researchers of marine teleosts find it difficult to establish an experimental environment such as a freshwater facility to breed and rear freshwater model teleosts, such as zebrafish or medaka and (3) researchers often consider that the direct analysis of the targeted fish species is more appropriate.

**Figure 1:**
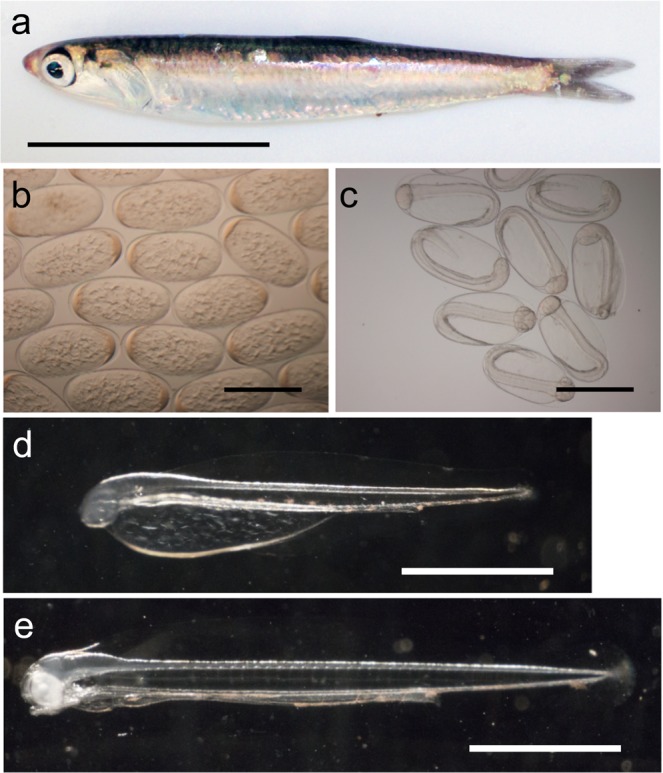
Japanese anchovy. (**a**) An adult fish. Scale bar: 5 cm. (**b**) Embryos shortly after fertilisation. Scale bar: 1 mm. (**c**) Embryos at 40 hpf (hours post-fertilisation; shortly before hatching). Scale bar: 1 mm. (**d**) An embryo at 48 hpf and (**e**) at 72 hpf. Scale bar: 10 mm. The embryos, larvae and fish were maintained and reared at approximately 20 °C throughout the study.

Therefore, we focused on the Japanese anchovy (*Engraulis japonicus*) as a candidate model organism for marine teleosts, which would be easy to handle for researchers of marine teleosts. The Japanese anchovy is a forage fish of the family Engraulidae; its body length is approximately 10–11 cm (Fig. 1a) and it has a lifespan of 2–3 years. The Japanese anchovy has almost every advantage required for a model organism, including the following: (1) it is readily obtainable; (2) it is easy to breed and maintain, with a complete aquaculture system using small-scale fish tanks having been established^2^; (3) it is prolific (each female spawns approximately 500 eggs per day at a water temperature of 20 °C and approximately 1,000 eggs per day at a water temperature of 25 °C; egg production can be predicted in relation to the temperature^2^); (4) the egg membrane is transparent; therefore, the embryonic development process can be readily observed *in situ* (Fig. 1b to e); (5) the embryonic development process is rapid (with hatching occurring approximately 41 h after fertilisation (hours post-fertilisation, hpf) at a water temperature of 20 °C) and (6) its generation time is short (it develops into a mature individual approximately 3 months after hatching). However, to establish the Japanese anchovy as a model organism, it is essential to establish a versatile experimental system including genetic modification technology and gene disruption methods in addition to the improvement of the gene information available by RNA-seq and genomic analysis.

Therefore, the present study established a basic, yet comprehensive experimental system for a marine teleost, the Japanese anchovy. This system, which includes various methodologies to establish the Japanese anchovy as a model organism, could facilitate the field of experimental biology of marine teleosts.

## Results

### Control of spawning time

**Figure 2:**
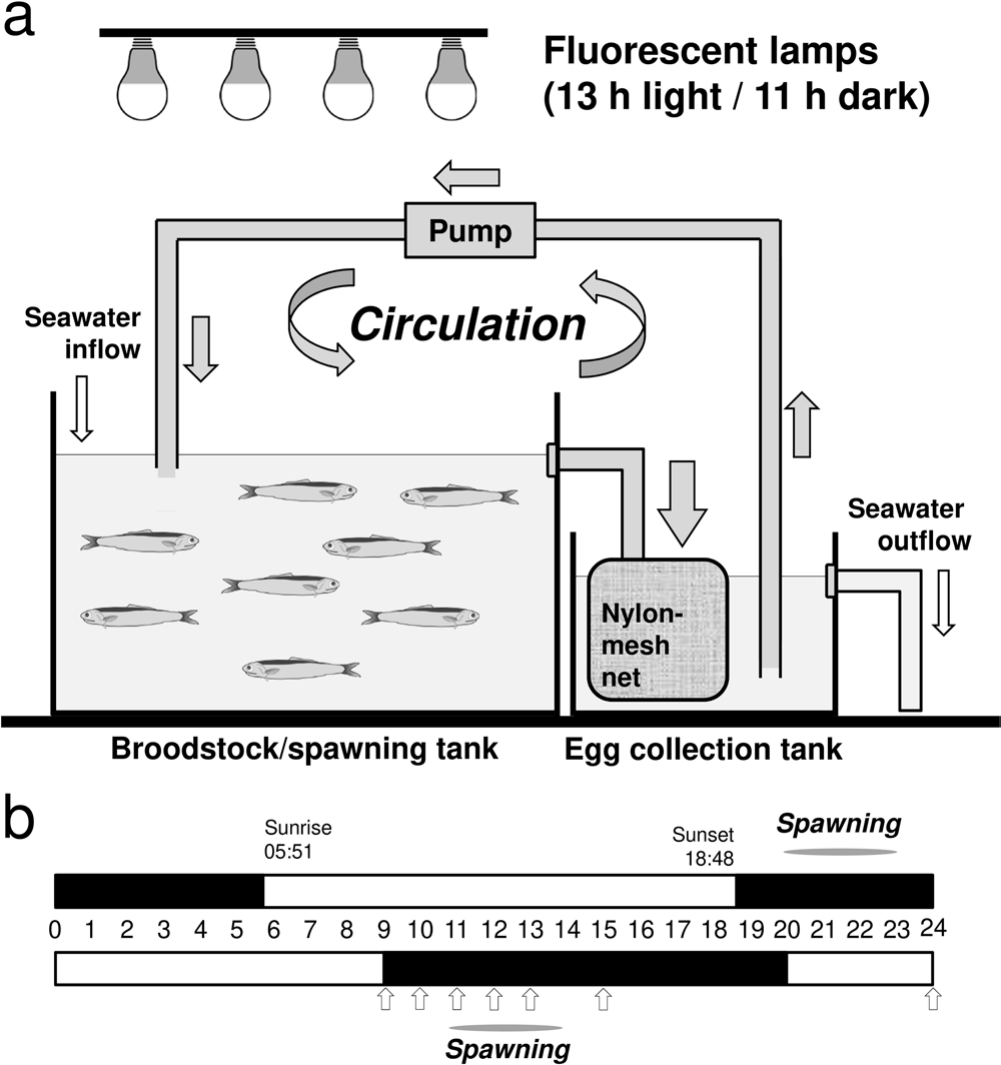
Spawning of the Japanese anchovy under artificial rearing conditions. (**a**) Schematic structure of the rearing/spawning facility of the Japanese anchovy. Arrows indicate the water flow direction. (**b**) Spawning time of the Japanese anchovy under natural (upper bar) or artificially reversed day-night photoperiod (lower bar). Black and open bars represent night and day times, respectively. Arrows indicate the sampling times of the eggs.

A total of 85 adult Japanese anchovies spawning every day under natural daylength and water temperature were transferred to the rearing/spawning facility designed by the present study (Fig. 2a, details described in ‘Methods’). The water temperature was kept constant at approximately 20 °C. The fishes were reared for 12 d under an artificial photoperiod where day and night were reversed (13 L:11D, lights were turned on between 20:00 and 09:00). The spawned eggs were collected at 1-h intervals between 09:00 and 13:00 and additional sampling was undertaken at 15:00 and 24:00 (Fig. 2b). The collection of eggs started at 11:00 and peaked around 13:00, whereas no eggs were collected at midnight (24:00), which is the principal spawning time under a natural photoperiod. The fertilised eggs at the one- and two-cell stages were collected at 11:00 and 12:00 (Table 1). Furthermore, the fishes continued to spawn every day throughout the year until the end of their lives. These results clearly indicate that the spawning of the Japanese anchovy can be readily controlled by regulating the photoperiod and the water temperature. The one-cell eggs obtained were used in subsequent experimentation.

**Table 1:** Spawning of Japanese anchovy under an artificial photoperiod.

Sampling hours	Approx. no. of eggs	Developmental stages
9:00	0	—
10:00	0	—
11:00	1,000	1 cell (shortly after spawning)
12:00	5,000	1–2 cells
13:00	9,500	Blastula
15:00	2,000	Blastula
24:00	0	—

### Establishment of microinjection system

**Figure 3:**
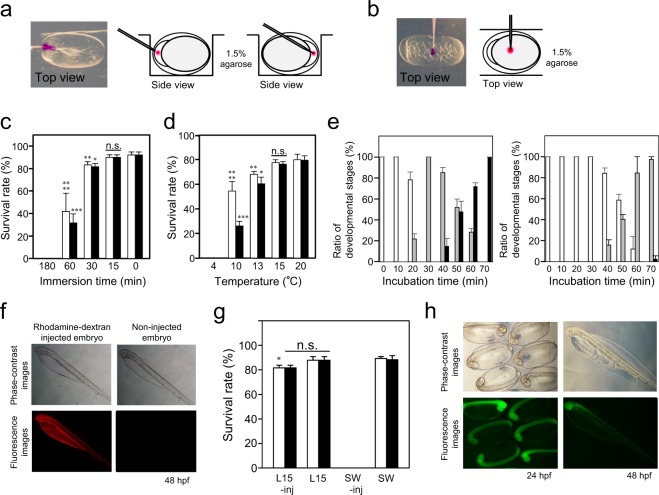
Microinjection of the Japanese anchovy embryos. (**a**) Cytoplasmic injection. Injection needle can penetrate either from the animal pole (left) or vegetal pole (right) side of the eggs. (**b**) Yolk injection. Injection solution (200 mM KCl containing 0.05% (*w/v*) rhodamine-dextran) was injected into the one-cell eggs under immersion in Leibovitz’s L-15 medium. (**c**) Influence of Leibovitz’s L-15 medium immersion on embryogenesis (n =≥ 15, three independent experiments). One-cell eggs were immersed in Leibovitz’s L-15 medium for different times as indicated, and their survival rate at 24 hpf (open bars) and 48 hpf (black bars) was observed. *p = 0.01, **p = 0.017957, ***p = 0.006581, ****p = 0.033942 and n.s. (not significant: p > 0.05) as compared with non-immersed control (0 min). (**d**) Influence of low-temperature treatment on embryogenesis (n =≥ 25, three independent experiments). One-cell eggs were incubated in seawater for 1 h at different water temperatures as indicated, and their survival rate at 24 hpf (open bars) and 48 hpf (black bars) was observed at 20 °C. *p = 0.007072, **p = 0.026275, ***p = 6.27 × 10^−5^, ****p = 0.016097 and n.s. (not significant: p > 0.05) as compared with non-treatment control (20 °C). (**e**) Influence of low-temperature treatment on the speed of early embryonic development. One-cell eggs were incubated in seawater at 20 °C (left) or 15 °C (right), and their developmental stages were observed at different incubation time points as indicated. White, grey and black bars represent one-cell, two-cell and four-cell eggs, respectively. (**f**) Images of the rhodamine-dextran-injected embryo. One-cell eggs were injected with 0.05% (*w/v*) rhodamine–dextran solution into the yolk, and their images were observed under a fluorescence microscope at 48 hpf. (**g**) Microinjection using Leibovitz’s L-15 medium as the injection medium. While one-cell eggs were immersed in Leibovitz’s L-15 medium or in seawater for 30 min at 15 °C, microinjection manipulations were either performed or not (n =≥ 18, three independent experiments). Their survival rate at 20 °C was observed at 24 hpf (open bars) and 48 hpf (black bars). *p = 0.007128 and n.s. (not significant: p > 0.05) as compared with non-treatment control (SW). L15-inj, injected under immersion in Leibovitz’s L-15 medium; L15, not injected under immersion in Leibovitz’s L-15 medium; SW-inj, injected under immersion in seawater; SW, not injected under immersion in seawater (control). (**h**) Images of the *GFP*-mRNA injected embryos. One-cell eggs were injected with *GFP* mRNA (100 ng/µL) into the yolk and their images were observed under a fluorescence microscope at 24 and 48 hpf. Values are expressed as the means ± S.D. Statistical analyses were performed using Welch’s t-test.

As shown in Fig. 3a,b, one-cell eggs of the Japanese anchovy, which have a rugby-ball shape, were arranged in the holes or grooves of a 1.5% agar plate filled with seawater and a solution (200 mM KCl containing 0.05% (*w/v*) rhodamine–dextran as tracer dye) was injected into the cytoplasm or yolk of the embryos. The injection manipulation was performed at 20 °C. The chorion of teleost eggs hardens with time following fertilisation and thus it is difficult to perform microinjection with a glass needle in many cases without either dechorionisation or the prevention of chorion hardening. The intact chorion of the Japanese anchovy could be easily penetrated using a glass needle with a specially sharpened edge (details described in ‘Methods’); however, all embryos died immediately (Fig. 3g). This was caused by the considerable difference between the internal and external osmotic pressures of the egg; therefore, the injection medium supporting the relevant osmotic pressure for embryos was investigated. In red seabream, *Pagrus major*, Leibovitz’s L-15 medium was used as the injection medium^3^, so we examined whether Leibovitz’s L-15 medium was applicable to the Japanese anchovy. After one-cell eggs were immersed in Leibovitz’s L-15 medium for various periods, their embryogenesis at 24 and 48 hpf were observed at 20 °C (embryos hatched out at 41 hpf). The survival rate of the embryos was scarcely affected up to 30 min; however, prolonged immersion caused high embryo mortality (Fig. 3c). We therefore determined that the injection manipulation should be performed within 30 min when Leibovitz’s L-15 medium is used as the injection medium.

It is necessary for the injection manipulation to be performed during early embryogenesis, in most cases in one-cell eggs shortly after spawning and fertilisation or, at the latest, in two-cell eggs after the first cleavage. To increase the number of embryos injected for each manipulation, the effect of a low-temperature treatment on the speed of early embryonic development was assessed. The embryos and their parent fishes were maintained and reared usually at 20 °C throughout the present study. Eggs were collected shortly after spawning and incubated in seawater at various temperatures for 1 h, and their survival rate at 24 and 48 hpf was observed. Embryos were not affected (i.e. survival was not significantly different from that at 20 °C) in the 15 °C treatment, whereas the survival rate decreased in the 13 °C treatment, while the 4 °C treatment resulted in the death of all embryos (Fig. 3d). Thus, early embryogenesis at 20 °C and 15 °C were compared. The appearance of four-cell eggs after the second cleavage, i.e. the upper limit time for injection manipulation, took approximately twice as long: 40 min at 20 °C and 70 min at 15 °C (Fig. 3e). Thus, the eggs could be subjected to injection manipulation for approximately ~70 min when they were incubated at 15 °C, so that the injection manipulation within 30 min could be performed twice at each egg sampling. Subsequently, we examined whether the materials injected into the yolk were transported into the cytoplasm via cytoplasmic streaming. The cytoplasmic streaming in a Japanese anchovy embryo at the one-cell stage is shown in Supplementary Fig. S1. Direct microinjection into the cytoplasm is more effective but requires careful orientation of the embryos and is more time-consuming. One-cell eggs were injected with 0.05% (*w/v*) rhodamine–dextran solution into the yolk and injection manipulation was performed at 15 °C in Leibovitz’s L-15 medium. Rhodamine–dextran in the yolk quickly moved into the cytoplasm via cytoplasmic streaming, and embryogenesis progressed normally (Fig. 3f). The survival rate of injected embryos at 24 and 48 hpf was similar to that of the non-injected controls (Fig. 3g).

To confirm the usability of the microinjection system, the embryos were injected with *GFP* mRNA and the fluorescence derived from GFP was observed. RNA (100 ng/µL) was injected into the egg yolk during the one-cell stage under immersion in Leibovitz’s L-15 medium. Eggs were maintained at 15 °C to delay embryogenesis and the injection was performed within 30 min to prevent a decrease in survival rates. The eggs were then transferred to seawater and incubated at 20 °C. Figure 3h shows the phase-contrast and fluorescence images of injected embryos at 24 and 48 hpf. Their embryogenesis progressed normally and strong whole-body GFP fluorescence was detected. The survival rates of injected and non-injected embryos at 48 hpf were 87.5% (28 in 32 embryos) and 90.0% (45 in 50 embryos), respectively.

### *Tol2* transposon-mediated transgenesis

**Figure 4:**
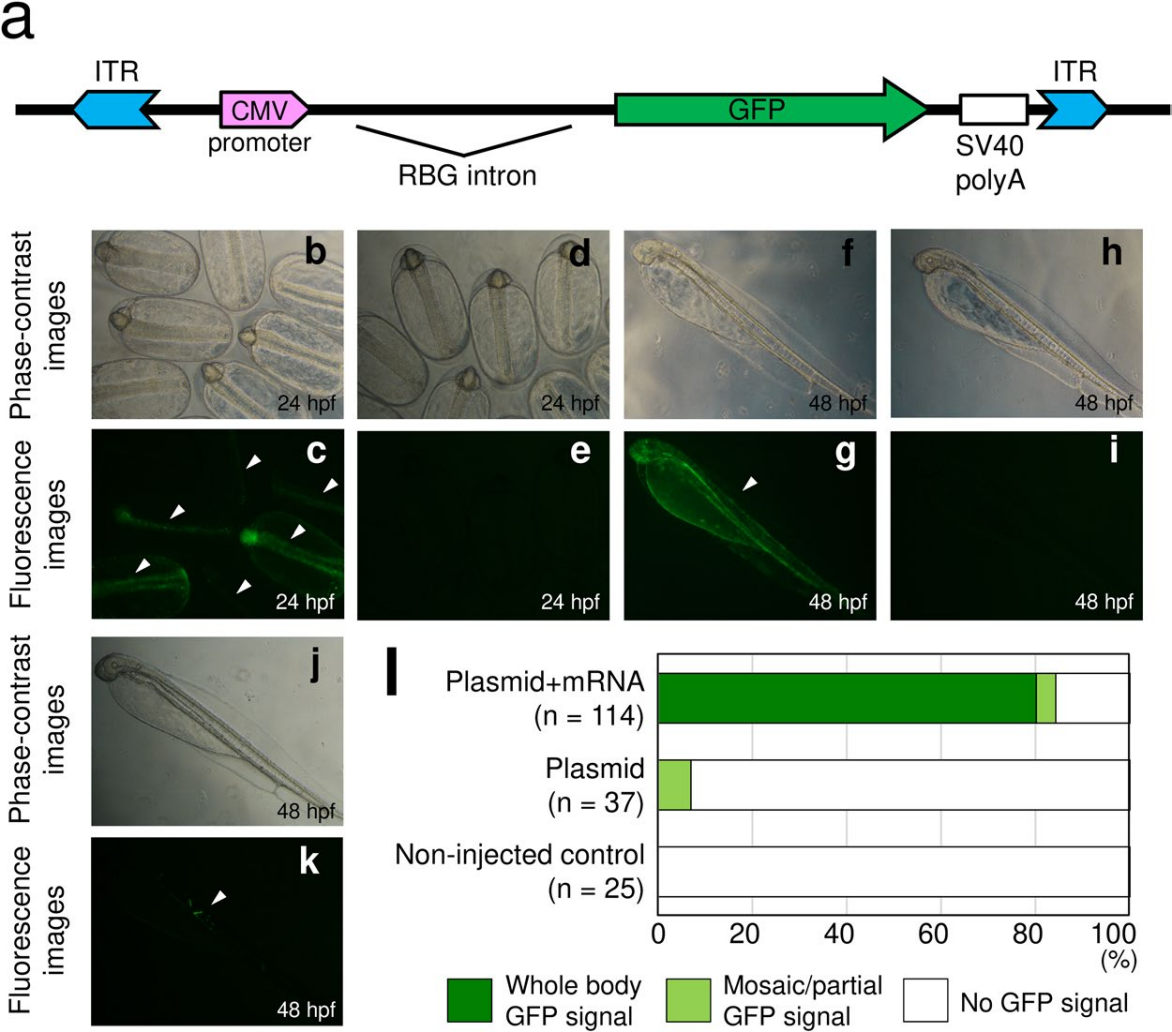
*Tol2*-mediated transgenesis in the Japanese anchovy embryos using *GFP* as the reporter gene. (**a**) Schematic diagram of pT2AL200R150CG. Abbreviations: ITR (Tol2 inverted terminal repeat), CMV (cytomegalovirus), GFP (green fluorescent protein), RGB intron (rabbit beta-globin intron), SV40 polyA (SV40 polyadenylation signal). (**b–k**) Images of *GFP*-gene-transfected embryos. (**b**,**c**,**f** and **g**) Plasmid/RNA co-injected embryos. (**j** and **k**) Plasmid-injected embryo. (**d**,**e**,**h** and **i**) non-injected embryos. The plasmid (12.5 ng/µL) was injected either with or without *Tol2* transposon mRNA (25 ng/µL) into the yolk of embryos during the one-cell stage, and their images were observed under a fluorescence microscope at 24 and 48 hpf. White arrowheads indicate GFP-positive embryos. (**l**) Efficiency of *Tol2*-mediated transgenesis. GFP-positive embryos were counted at 48 hpf.

The *Tol2* transposable element from medaka fish (*O. latipes*)^4^ mediates a high rate of foreign DNA integration into the genome of various vertebrates, from fish to mammals^5^. Here, we tested whether the *Tol2* system worked effectively in the Japanese anchovy by conducting transient transgenesis experiments. A plasmid comprising the cytomegalovirus (CMV) promoter region and the *GFP* reporter gene, flanked by the *Tol2* insertion sites, was constructed and termed pT2AL200R150CG (Fig. 4a). The plasmid (12.5 ng/µL) was injected either with or without *Tol2* transposon mRNA (25 ng/µL) into the yolk of embryos at the one-cell stage. Embryos were then observed under a fluorescence microscope at 24 and 48 hpf. Many embryos (80.7%, 92 of 114 embryos) that were co-injected with both the plasmid and mRNA showed whole-body GFP fluorescence (Fig. 4c,g). By contrast, mosaic/partial GFP fluorescence was observed in only a few embryos (5.4%, 2 of 37 embryos) when the plasmid only was injected (Fig. 4k). No GFP signal was detected in the non-injected control (Fig. 4e,i). These results, summarised in Fig. 4l, revealed that the *GFP* gene was integrated into the somatic genome of the Japanese anchovy with high efficiency by *Tol2* transposon-mediated gene transfer. Furthermore, the same results were obtained when introducing the plasmid with the DsRed reporter gene driven by the *Xenopus* EF-1 α enhancer/promoter (Supplementary Fig. S2).

### Transcription Activator-Like Effector Nucleases (TALEN)- and Clustered Regulatory Interspaced Short Palindromic Repeats (CRISPR)/Cas9-mediated genome editing

**Figure 5:**
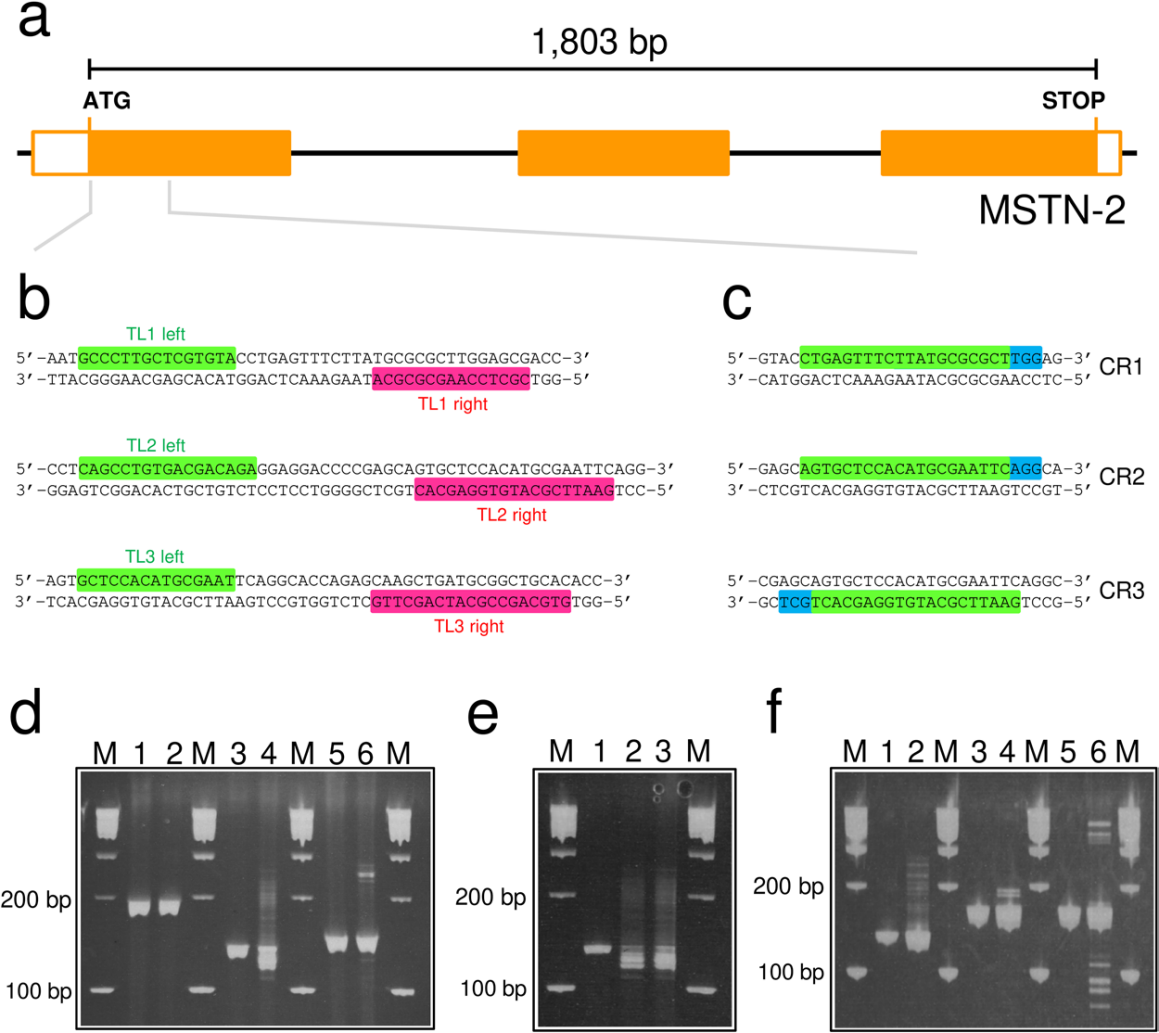
Genome editing in the Japanese anchovy. (**a**) Schematic representation of the genomic structure of the myostatin-2 (*MSTN-2*) gene. Coding and untranslated exon regions are shown as solid and open boxes, respectively. (**b**) Design of TALENs. Three TALENs termed TL1, TL2 and TL3 were designed using TAL Effector-Nucleotide Targetter 2.0 (https://tale-nt.cac.cornell.edu/node/add/talen)^26^. Green and pink boxes indicate the left and right recognition sequences of the TALENs, respectively. (**c**) Design of gRNA for CRISPR/Cas9. Three gRNAs termed CR1, CR2 and CR3 were designed with the GeneArt CRISPR Search and Design Tool (Thermo Fisher Scientific). The gRNA targeting sequence and Protospacer Adjacent Motif are indicated by green and blue boxes, respectively. (**d**) The heteroduplex mobility assay (HMA) profiles of embryos injected with TALEN mRNAs. One-cell eggs were injected with TALEN mRNA pairs (150 ng/μL each). The HMA was performed using a 15% polyacrylamide gel. Lanes: M, 100 bp marker; 1, non-injected control for TL1; 2, TL1-injected embryos; 3, non-injected control for TL2; 4, TL2-injected embryos; 5, non-injected control for TL3; 6, TL3-injected embryos. (**e**) The HMA profiles of TL2-injected embryos by the yolk or cytoplasm injection. One-cell eggs were injected with TL2-mRNA pairs (150 ng/μL each) into the yolk or the cytoplasm. The HMA was performed as described in (**d**). Lanes: M, 100 bp marker; 1, non-injected control; 2, yolk-injected embryos; 3, cytoplasm-injected embryos. (**f**) The HMA profiles of embryos injected with gRNAs and Cas9 protein. One-cell eggs were injected with gRNAs and Cas9 nuclease protein (600 and 200 ng/µL, respectively), which were separately co-injected into the yolk and subjected to HMA as described in (**d**). Lanes: M, 100 bp marker; 1, non-injected control for CR1; 2, CR1-injected embryos; 3, non-injected control for CR2; 4, CR2-injected embryos; 5, non-injected control for CR3; 6, CR3-injected embryos.

Genome editing using TALEN^6^ and CRISPR/Cas9^7^ has attracted considerable attention recently because these technologies can achieve targeted and efficient genetic modifications (e.g. knockout, knock-in and so on). To establish a genome-editing system in the Japanese anchovy, we examined targeted mutagenesis at a specific gene locus by both TALEN- and CRISPR/Cas9-mediated genome-editing technologies. Initially, three TALEN pairs targeting the myostatin-2 (*MSTN-2*) gene were designed (termed TL1, 2 and 3; Fig. 5a,b) and the *in vitro* transcribed RNAs of the pairs (150 ng/µL each) were separately co-injected into the embryo yolk at the one-cell stage. Genomic DNAs were extracted from five embryos in each experiment at 21 hpf and subjected to the heteroduplex mobility assay (HMA)^8^ to detect TALEN *in vivo* activity. TALEN-induced insertion and/or deletion (indel) mutations were estimated by the degree of the multiple HMA profiles. Strong and weak TALEN activities were observed in TL2- and TL3-injected embryos, respectively, but no TALEN activity was detected in TL1-injected embryos (Fig. 5d). No apparent difference in the HMA profiles between the yolk and cytoplasm injections was observed in the TL2-injected embryos (Fig. 5e). Amplicon deep-sequencing analysis of the TL2-target sequence of the yolk-injected embryos (21 hpf, n = 10) showed that the indel frequency was identified as being extremely high, i.e. 22,079 in 23,624 sequenced clones (93.5%) had altered sequences consisting of 106 types of mutations. The frameshift-mutation rate was 43.0% (10,146 in 23,624 clones) consisting of 58 types of mutations. The multiple alignment of mutation sequences is shown in Supplementary Fig. S3. Subsequently, three CRISPR guide RNAs (gRNAs) targeting *MSTN-2* were designed (termed CR1, 2 and 3; Fig. 5a,c) and each gRNA and Cas9 nuclease protein (600 and 200 ng/µL, respectively) were separately co-injected into the cytoplasm of embryos at the one-cell stage. As shown in Fig. 5f, all gRNAs tested showed some mutagenic activity.

### Production of genetically modified Japanese anchovy

**Figure 6:**
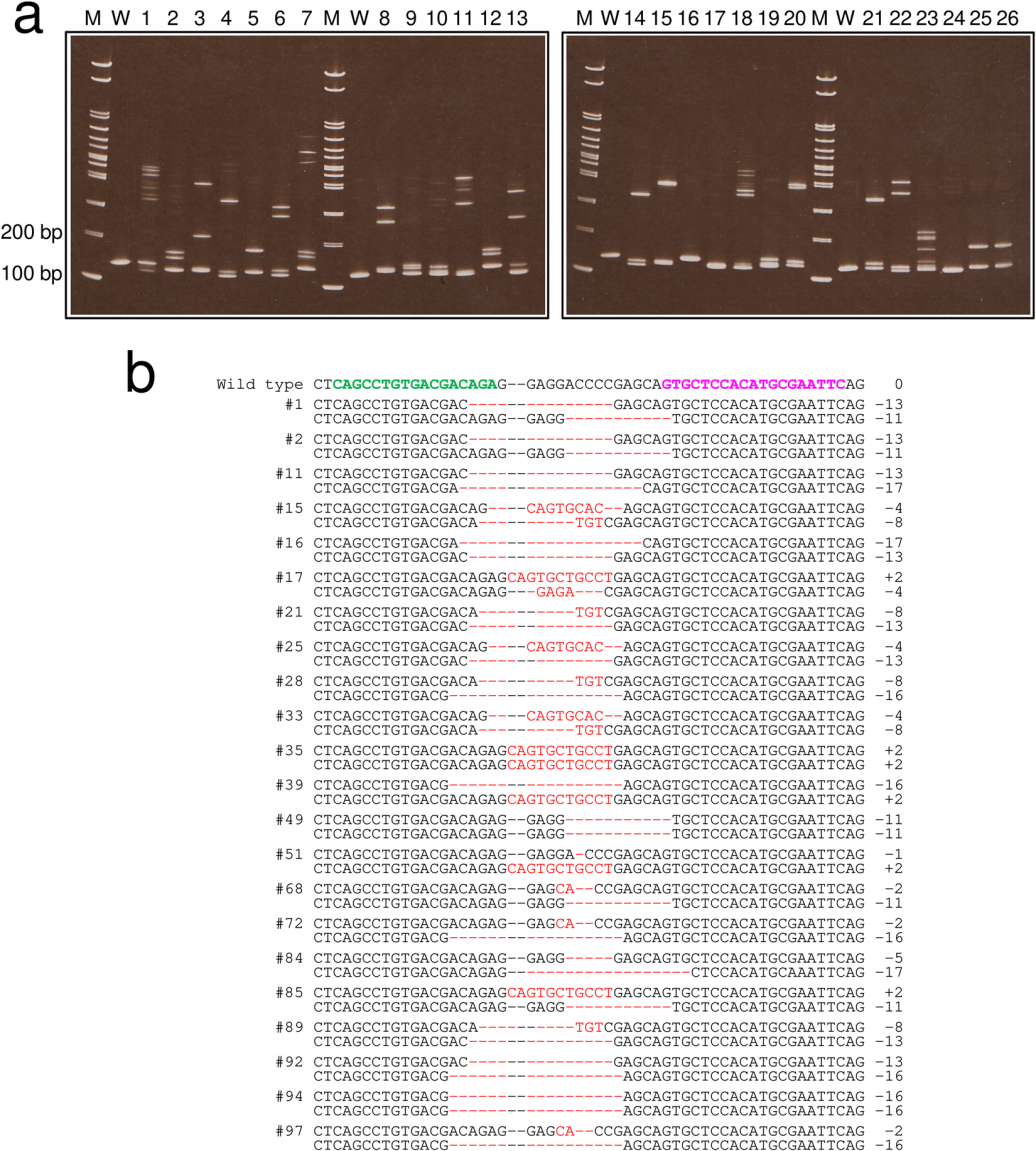
Establishment of the *MSTN-2*^−/−^ lineage. (**a**) The HMA profiles of F_1_ individuals. The genomic DNAs were extracted from caudal fin clips of 26 F_1_ individuals and the HMA was performed using two 10–20% gradient polyacrylamide gels. Lanes: M, 100 bp marker; W, non-injected control; numbers, F_1_ individual number. (**b**) Frameshift-mutation sequences in F_1_ individuals. The DNA fragment including the TL2-target sequence was amplified by PCR. The amplicons were subcloned and sequenced. This is shown in order from the left: F_1_ individual number; sequences; the sizes of the insertions and deletions (−, deletions; +, insertions). Green and pink letters indicate the left and right recognition sequences of the TALENs, respectively. Red dashes and letters indicate the identified mutations.

To establish genetically modified animals, whether by stable transgenesis or genome editing (except for CRISPR/Cas9-mediated gene drive^9^), it is generally necessary to select the individuals with the genotype of interest and to breed them over generations. The first influential factor affecting the difficulty of the process is the germline transmission efficiency in F_0_ mosaic animals and, therefore, we aimed to establish the *MSTN-2* knockout (*MSTN-2*^−/−^) lineage as a practical example of genetically modified Japanese anchovy. TL2-mRNA pairs (Fig. 5b), which showed the very high mutagenic activity mentioned above, were injected into the yolk of one-cell eggs; the F_0_ individuals were reared to sexual maturity. A total of 1,165 eggs were microinjected, of which 867 embryos hatched out normally and 99 individuals grew into adult fishes. The F_0_ founders were then crossed with each other to produce F_1_ offspring. HMA profiles showed that 24 of 26 F_1_ individuals (92.3%) had mutations at the *MSTN-2* gene locus (Fig. 6a, lanes: 1–15, 17–23, 25 and 26), indicating that the germline transmission rate was sufficiently high. The genotyping analysis revealed that 22 in 104 F_1_ individuals (21.2%) had frameshift mutations in both *MSTN-2* alleles consisting of 12 types of mutations (Fig. 6b). Therefore, *MSTN-2*^−/−^ fishes were established in the F_1_ generation. At present, the F_1_ progeny have been reared to the F_4_ generation and their *MSTN-2*^−/−^ genotypes have been confirmed.

## Discussion

The aim of the present study was to establish a model organism that was manageable for researchers studying marine teleosts. Tiger pufferfish, *Takifugu rubripes*, is a well-known marine teleost that is available as a model organism for genomic studies because of its compact genome; however, it is not suitable for experimental biology studies owing to the difficulty in its mass rearing and its long generation time (the age for male and female sexual maturity is 2 and 3 years, respectively^10^). Therefore, we developed a comprehensive experimental system for the Japanese anchovy. The system is composed of methodologies for rearing, egg collection, microinjection, gene manipulation and breeding; that is, it includes basic technologies to undertake experiments using the Japanese anchovy as a model organism. In addition, transcriptome and genome analysis of Japanese anchovy is now in progress.

To perform microinjection, fertilised eggs at the one-cell stage prior to first cleavage are required. A rearing/spawning facility that regulates the photoperiod and water temperature was constructed (Fig. 2a); consequently, we obtained one-cell eggs shortly after spawning at the required times (Fig. 2b and Table 1). This technique, which is widely used in zebrafish and medaka, also contributed considerably to the success of the present study.

The *Tol2* transposon mediates efficient foreign DNA integration into vertebrate genomes and is now widely used as a genetic tool for transgenesis, insertional mutagenesis, gene trapping and enhancer trapping^11^. In teleosts, the use of the *Tol2* system in zebrafish is widespread in various fields of life science. However, its use in other teleosts has been limited to some fishes, namely three-spined stickleback (*Gasterosteus aculeatus*)^12^, haplochromine cichlid (*Astatotilapia burtoni*)^13^, African turquoise killifish (*Nothobranchius furzeri*)^14^, the Nile tilapia (*Oreochromis niloticus*)^15^ and Midas cichlid (*Amphilophus citrinellus*)^16^. In the present study, *Tol2*-mediated transgenesis was successfully performed in the Japanese anchovy (Fig. 4). To the best of our knowledge, this is the first study in which the *Tol2* system has been shown to work in marine teleosts.

MSTN, also called the growth and differentiation factor 8, is a member of the transforming growth factor-β superfamily, and it functions as a negative regulator of skeletal muscle growth. Gene targeting of an *MSTN* gene in mice produced a double-muscle phenotype with increased skeletal muscle mass resulting from both muscle fibre hyperplasia and hypertrophy^17,18^. Naturally occurring *MSTN* null-mutant cattle breeds^19^, such as Piedmontese and Belgian Blue, are used as beef cattle with markedly increased muscle production. In teleosts, many previous studies had been conducted to elucidate the function of *MSTN* and to increase the edible portion of the fish by the loss of function of the *MSTN* gene. However, most teleosts, including the Japanese anchovy, possess at least two *MSTN* paralogs^20^ (Supplementary Fig. S4) and their functional divergence has not yet been clarified. We established a *MSTN-2*^−/−^ lineage for the Japanese anchovy (Fig. 6), and the objective of future study is to perform comparative studies of *MSTN-1*^−/−^, *MSTN-2*^−/−^ and *MSTN-1*^−/−^ /*MSTN-2*^−/−^ (double knockout) lineages.

In conclusion, we established a comprehensive experimental system for the Japanese anchovy (*E. japonicus*), which is a promising candidate for use as a model organism for marine teleosts. It is possible that the system can be applicable, with some modifications, to related species such as the Peruvian anchoveta (*Engraulis ringens*), European anchovy (*Engraulis encrasicolus*), Southern African anchovy (*Engraulis capensis*) and Californian anchovy (*Engraulis mordax*). We hope that using these anchovies as model organisms will aid the progress of experimental biology research in marine teleosts.

## Methods

### Animals

Japanese anchovies (*E. japonicus*) from Ohmura Bay at Nagasaki, Japan, were purchased from a fishery company (Takeshita Suisan, Nagasaki, Japan) and transferred to the laboratory of the Fisheries Research Institute of Karatsu of Kyushu University (Saga, Japan). They were kept in circular tanks (1- or 3-tonne tanks) and fed with 5–7% of their body weight of artificial diet (Ezuke-ru 0; Chubu Shiryo) 3–5 times per day. Seed production procedure is described in Supplementary Methods. All animal experiments were performed according to the guidelines of the Animal Experiments Committee at Kyushu University; all experimental protocols were endorsed by the committee.

### The rearing/spawning facility

The rearing/spawning facility comprised two large and small volume circular tanks (i.e., 1- and 0.2-tonn tanks); the former was a broodstock/spawning tank and the latter was an egg collection tank (Fig. 2a). The two tanks were placed in close contact with each other and a magnetic drive pump was installed between them to maintain an adequate quantity of circulating water. The spawned eggs were efficiently collected from a net attached to the drain outlet of the rearing/spawning tank. The photoperiod and water temperature were artificially controlled throughout the year: the photoperiod was set at 13-h of light and 11-h of darkness (13 L:11D) using cool-white fluorescent lamps, with the intensity of lights at the water surface being approximately 500 lux, while the water temperature was kept constant at approximately 20 °C.

### Embryonic culture

Shortly after spawning, eggs were collected and left to stand for a few minutes to distinguish between fertilised (living) eggs and unfertilised (dead) eggs; fertilised eggs float, whereas unfertilised eggs sink to the bottom. The floating eggs on the water surface were carefully withdrawn using a plastic pipette; groups of ~50 eggs each were transferred to a plastic Petri dish (15-cm diameter), filled with sterile seawater passed through a 0.22-µm filter and incubated at 20 °C for up to 4 d. To maintain water quality, dead embryos were removed and the seawater was replaced after 3 and 24 h incubation. The embryos were observed under stereomicroscope (Leica M60; Leica Microsystems); their images were captured using digital camera (EOS Kiss X6i; Canon Inc.).

### Microinjection

Microneedles were made from a cored glass tube (GD-1; Narishige) using a Micropipette Puller PC-10 (Narishige). The needle tips were ground to a 35° angle using a Micro Grinder EG-400 (Narishige) to produce a tip diameter of approximately 1–2 µm. A sharp edge on the needle tip was made using Micro Forge MF-900 (Narishige) to easily penetrate the chorion of intact fertilised eggs. Shortly before injection, the needle was backfilled with ~5 µL of injection solution containing 0.05% (*w/v*) phenol red (Sigma-Aldrich) as the tracer dye using an Eppendorf Microloader pipette tip (Eppendorf). The needle was set to an injection holder (HI-7; Narishige) that was fixed to a micromanipulator (MMO-220A and M-152; Narishige) beside a stereomicroscope (Leica M60; Leica); the injection holder was connected to a microinjector (IM-6; Narishige). Shortly after spawning, fertilised eggs were preincubated at 15 °C in a 15-cm diameter plastic Petri dish filled with filtered seawater to delay embryonic development until subsequent use. The eggs were transferred to a 6-cm diameter plastic Petri dish filled with Leibovitz’s L-15 medium and carefully arranged in the holes or grooves of a 1.5% agar plate (prepared by placing a plastic mould on the agarose before solidification; Fig. 3a,b) using fine tip pipettes under stereomicroscope observation. To avoid decreases in the embryonic survival rates, injection manipulation was performed within 30 min of suspension in Leibovitz’s L-15 medium. Thereafter, injected eggs were immediately transferred to filtered seawater in a 15-cm diameter plastic Petri dish and incubated at 20 °C.

### *In vitro* synthesis of *GFP* mRNA and microinjection

The *GFP* gene that incorporated a T7 promoter sequence at the 5′end was amplified by polymerase chain reaction (PCR) using pAcGFP1-1 (clontech) as the template and subjected to *in vitro* transcription using a mMESSAGE mMACHINE T7 Transcription Kit (Thermo Fisher Scientific). Supplementary Table S1 lists the PCR primers used. The resultant capped *GFP* mRNA was purified with an RNeasy Mini Kit (Qiagen). Prior to microinjection, the *GFP* mRNA was diluted to a final concentration of 100 ng/µL in DNase-/RNase-free water containing 0.05% (*w/v*) phenol red (Sigma-Aldrich). Embryos were injected with the *GFP* mRNA into the yolk at the one-cell stage.

### Fluorescence microscopy

Embryos that hatched were anaesthetised with 0.01% (*w/v*) benzocaine (Sigma-Aldrich) in Ringer’s solution for marine fish (1.35% NaCl, 0.06% KCl, 0.025% CaCl_2_, 0.02% NaHCO_3_ and 0.035% MgCl_2_, *w/v*)^21^ and immobilised by placing them on 3% (*w/v*) methylcellulose in a 3.5-cm diameter plastic Petri dish. Phase-difference and fluorescence images were captured using Nikon DS-L3 camera (Nikon) mounted on a Leica DM IL LED (Leica Microsystems).

### Construction of *GFP* and DsRed expression vectors and microinjection

The CMV promoter region was amplified via PCR using pVenus-N1 as the template and ligated in the same site of pT2AL200R150G^22^. The resultant plasmid was designated pT2AL200R150CG (Fig. 4a). pTG-ds-miR-shGFP^23^ was digested with XbaI and EcoRI and the termini were blunted using DNA Blunting Kit (TaKaRa Bio). The DNA fragment was self-ligated; the resultant plasmid was designated pXef-DsRed (Supplementary Fig. S2a). The DNA fragment containing the SV40 poly(A) signal was prepared by digesting pCS2TAL3-DD^24^ with XbaI and NotI and was ligated in the same site of T7-TPase^25^. The resultant plasmid was designated pT7-TP. pT7-TP was linearised with NotI (Nippon gene) and used as the template for *in vitro* transcription using a mMESSAGE mMACHINE T7 Transcription Kit (Thermo Fisher Scientific). The resultant capped mRNA was purified with an RNeasy Mini Kit (Qiagen). Embryos were injected with a solution comprising the expression vector (12.5 ng/µL), *Tol2* transposase mRNA (25 ng/μl), RNase-free water and phenol red (0.05%, *w/v*) into the yolk during the one-cell stage.

### Construction of TALEN plasmids and microinjection

TALE repeat structures for *MSTN-2* were designed using TAL Effector-Nucleotide Targeter 2.0 (https://tale-nt.cac.cornell.edu/node/add/talen)^26^ with the following parameters: 14–17 bp for spacer length, 15–18 bp for repeat array length and only T in the upstream base. TALENs were assembled by the Golden Gate assembly method^27^ and the repeat arrays comprising 3–6 modules were obtained. Thereafter, both the repeat arrays and the last repeat modules were cloned into the expression plasmids pCSTAL3DD or pCSTAL3RR^24^. The target sites of TALENs are shown in Fig. 5a,b. The expression plasmids were then linearised with NotI (Nippon Gene) and used as the template for *in vitro* transcription using a mMESSAGE mMACHINE SP6 Kit (Life Technologies). The resultant capped mRNAs were purified with an RNeasy Mini Kit (Qiagen). Embryos were injected with a solution comprising the mRNAs (150 ng/μL each), RNase-free water and phenol red (0.05%, *w/v*) into the yolk at the one-cell stage.

### CRISPR/Cas9 microinjection

The PCR primers used for gRNA synthesis (Supplementary Table S1) were designed with the GeneArt CRISPR Search and Design Tool (Thermo Fisher Scientific). The gRNAs were synthesised using the GeneArt Precision gRNA Synthesis Kit (Thermo Fisher Scientific). The gRNA and GeneArt Platinum Cas9 nuclease (200 ng/µL and 600 ng/ µL, respectively, each in 0.05% phenol red) were co-injected into the cytoplasm of embryos at the one-cell stage.

### Heteroduplex mobility assay

Samples (embryos, larvae or caudal fin clips) were lysed in lysis buffer (10 mM Tris-HCl, pH 8.0, 1 mM EDTA, 0.3% Tween-20, 0.3% NP-40, 0.2 mg/mL proteinase K) for 5 h at 55 °C. After inactivation of proteinase K at 95 °C for 15 min, a short fragment including the target site of TALENs or CRISPR/Cas9 was amplified by PCR using the lysates as the templates. The resulting amplicons were electrophoresed on either 15% polyacrylamide gels or 10–20% gradient polyacrylamide gels (Wako Pure Chemical Industries, Ltd.). Supplementary Table S1 lists the PCR primers used.

### Amplicon deep-sequencing

The DNA fragments, including the target site of TALEN TL2, were amplified by PCR using the lysate of TL2-injected embryos (21 hpf, n = 10) as the template and subjected to amplicon deep-sequencing. The detailed procedure is described in Supplemental Methods.

### Founder screening

Embryos were injected with TL2-mRNA pairs (150 ng/μL each; Fig. 5b) and the F_0_ adult fish obtained were mated with each other in the rearing/spawning facility. The F_1_ or later generations were subjected to genotyping analysis as follows: fish were anaesthetised with 300–500 ppm of 2-phenoxyethanol (Wako Pure Chemical Industries) in seawater. After tagging with Visible Implant Elastomer (Northwest Marine Technology, Inc.), the caudal fins were cut with a sharp scalpel and fish were quickly returned to a 0.2- or 0.5-tonne tank and reared until their genotypes were identified. To detect indel mutations, HMA was performed and the amplicons containing the target site were sequenced. Fish that were homozygous for the *MSTN-2* null mutation were selected and their offspring reared.

### Statistical analyses

All data are presented as mean ± standard deviation. The significance of differences between two dependent groups was assessed using two-tailed Welch’s t-test. P values for each test, the number of samples and independent experiments are indicated in each figure legend. P < 0.05 was considered statistically significant.

## Supplementary information

Title page, Supplementary Methods, Supplementary Table S1, Supplementary Figure S1, Supplementary Figure S2, Supplementary Figure S3, Supplementary Figure S4, Supplementary Reference

## Data Availability

All data generated or analysed during this study are included in this article (and in the Supplementary information file).
