# Supplementary material for: Comprehensive Experimental System for a Promising Model Organism Candidate for Marine Teleosts
Source: Sci Rep. 2019 Mar 20;9:4948. doi: 10.1038/s41598-019-41468-8 (PMC6426966; doi:10.1038/s41598-019-41468-8)
Supplement: Supplementary file 1 — Title page, Supplementary Methods, Supplementary Table S1, Supplementary Figure S1, Supplementary Figure S2, Supplementary Figure S3, Supplementary Figure S4, Supplementary Reference [file 41598_2019_41468_MOESM1_ESM.pdf]

Comprehensive Experimental System for a Promising Model Organism Candidate for Marine Teleosts.

Keishi Sakaguchi[1*], Michio Yoneda[2], Noriyoshi Sakai[3,4], Kanako Nakashima[5], Hajime Kitano[1], and Michiya Matsuyama[5].

[1]Fisheries Research Institute of Karatsu, Department of Joint Research, Faculty of Agriculture, Kyushu University, Saga 847-0132, Japan; [2]National Research Institute of Fisheries and Environment of Inland Sea, Hakatajima Station, Imabari 794-2305, Japan; [3]Genetic Strains Research Center, National Institute of Genetics, Mishima 411-8540, Japan; [4]Department of Genetics, School of Life Sciences, SOKENDAI (The Graduate University for Advanced Studies), Mishima 411-8540, Japan; [5]Laboratory of Marine Biology, Department of Bioresource Sciences, Faculty of Agriculture, Kyushu University, Fukuoka 819-0385, Japan.

***Corresponding author**
Address: Fisheries Research Institute of Karatsu, Department of Joint Research, Faculty of Agriculture, Kyushu University, 59-2 Oka, Karatsu-shi, Saga 847-0132, Japan.
Tel: +81-995-51-7470
Fax: +81-955-79-0022
E-mail: keishi_s@agr.kyushu-u.ac.jp

Supplementary Methods

Seed production. Fertilised eggs were transferred to smaller circular tanks (0.5- or 1-tonne tanks) at a stocking density of 10,000–15,000 eggs/tonne. Larvae and juveniles were fed an adequate supply of live S-type rotifers twice a day during 2–25 days after hatching (dah) and live brine shrimp twice a day during 15–50 dah, while artificial diets were fed from 30 dah.

Amplicon deep-sequencing. The DNA fragments including the target site of TALEN TL2 were amplified by PCR using the lysate of TL2-injected embryos (21 hpf, n = 10) as the template. The PCR primers used were as follows: primers NGS f1 (5′-TCG TCG GCA GCG TCA GAT GTG TAT AAG AGA CAG AGG GAA CCA CCA CAA CCA TCA CAC ACA CAC -3′) and NGS r1 (5′-GTC TCG TGG GCT CGG AGA TGT GTA TAA GAG ACA GGT GAT GGA TAT ATA CCC AGT GCG TAA AAC TTA CGC T-3′). The resulting amplicons were gel purified; the libraries were prepared following 16S Metagenomic Sequencing Library Preparation Protocol (Illumina). Sequencing was performed using Illumina Miseq Sequencer with 2 × 301 cycle Miseq Reagent Kit v3 (Illumina). Data analysis was performed as follows: the adapter sequences and low-quality regions were trimmed by Cutadapt-1.1 and Trimmomatic-0.32, respectively, paired-end sequence reads were assembled using the script fastq-join-1.1.2-537 and the read counts were calculated using an in-house script.

Supplementary Table S1. PCR primers used in this study.

In vitro synthesis of GFP mRNA	
**Primer name**	**Sequence (5'-3')**
AcGFPT7 F	GGT ACC TAA TAC GAC TCA CTA TAG GGT CGC CAC GGT GAG CAA GGG CGC CGA GCT GTT; T7 promoter sequence is underlined
AcGFPSV40PA R	GCA GTG AAA AAA ATG CTT TAT TTG TGA AAT G
gRNA synthesis	
**Primer name**	**Sequence (5'-3')**
CRP2 F1	TAA TAC GAC TCA CTA TAG AGT GCT CCA CAT GCG A
CRP2 R2	TTC TAG CTC TAA AAC GAA TTC GCA TGT GGA GCA C
CRP3 F1	TAA TAC GAC TCA CTA TAG CTG AGT TTC TTA TGC G
CRP3 R2	TTC TAG CTC TAA AAC AGC GCG CAT AAG AAA CTC A
CRP4 F1	TAA TAC GAC TCA CTA TAG GTC TTT TCC TAG CAG T
CRP4 R1	TTC TAG CTC TAA AAC GCG ACT GCT AGG AAA AGA C
HMA analysis	
**Target Primer name**	**Sequence (5'-3')**
Talen TL1, myo 34f	TAA CCT GCC GAA TGA ACA TAG TAC TAT AGC CA
Talen TL1, myo 286r	GTG TGC AGC CGC ATC AGC TT
Talen TL2, myo 36f	GCT TGG AGC GAC CTG TTT GGG CAA TTC CAC CAC A
Talen TL1, myo 169r	TCT GGG ACT TGA TGG TGT GCA GCC GCA TCA GCT T
Talen TL3, myo 48f	CTG TTT GGG CAA TTC CAC CAC ACC T
Talen TL3, myo 286r	GTG TGC AGC CGC ATC AGC TT
CHRISPR/Cas9 CR1, myo36f	GCT TGG AGC GAC CTG TTT GGG CAA TTC CAC CAC A
CHRISPR/Cas9 CR1, myo169r	TCT GGG ACT TGA TGG TGT GCA GCC GCA TCA GCT T
CHRISPR/Cas9 CR2, myo 34f	TAA CCT GCC GAA TGA ACA TAG TAC TAT AGC CA
CHRISPR/Cas9 CR2, myo 286r	GTG TGC AGC CGC ATC AGC TT
CHRISPR/Cas9 CR3, myo190f2	CAG ATC TTG AGC ATC CTC CGC CTT G
CHRISPR/Cas9 CR3, myo ampr1	GTT GTG GCG AGT TCC CCA TCG T

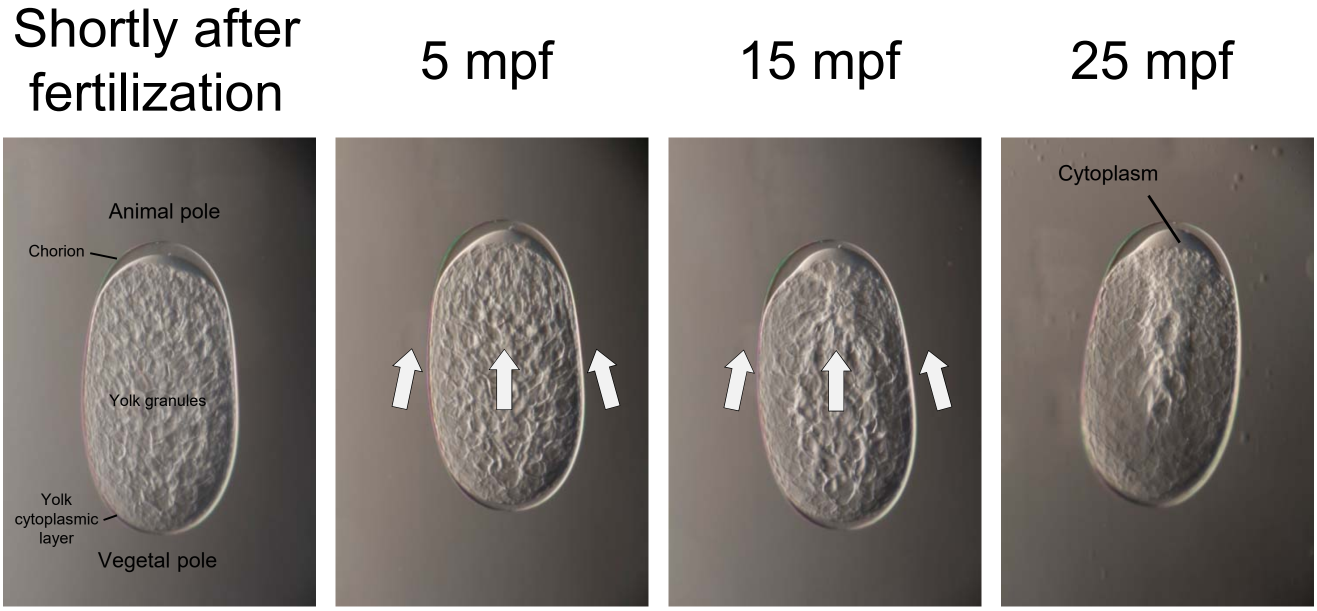

Supplementary Figure S1. Cytoplasmic streaming in Japanese anchovy embryo. Bright-field images of a Japanese anchovy embryo at one-cell stage. Arrows indicate the direction of cytoplasmic streaming. Abbreviations: mpf (minutes post-fertilisation).

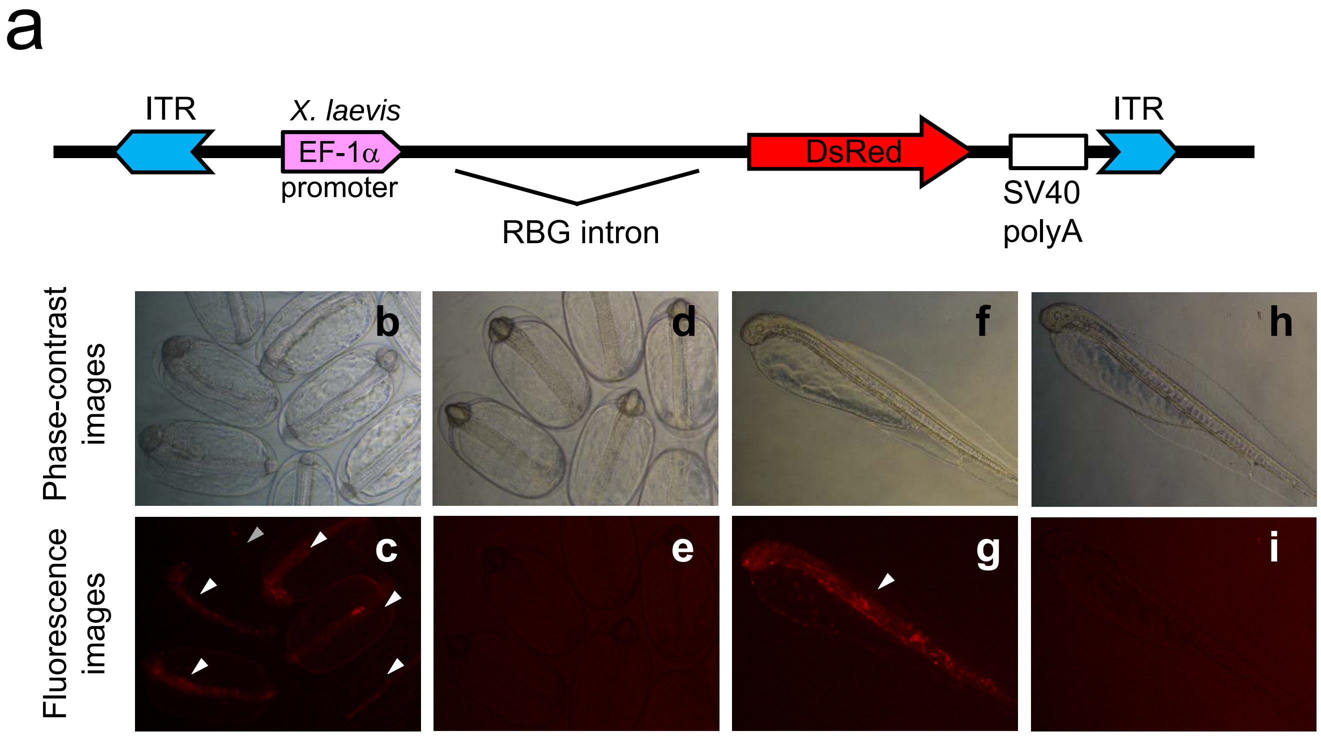

Supplementary Figure S2. Tol2-mediated transgenesis in Japanese anchovy embryos using DsRed as the reporter gene.

(**a**) Schematic diagram of pXef-DsRed. Abbreviations: ITR (Tol2 inverted terminal repeat), EF-1α (elongation factor-1 alpha), DsRed (*Discosoma* sp. Red Fluorescent Protein), RGB intron (rabbit beta-globin intron), SV40(A) (SV40 polyadenylation signal). (**b–i**) Images of DsRed gene-transfected embryos. (**b, c, f and g**) Plasmid/RNA-injected embryos. (**d, e, h and i**) non-injected embryos. The plasmid (12.5 ng/μL) and *Tol2* transposon mRNA (25 ng/μL) were injected into the yolk of embryos during the one-cell stage, and their images were observed under a fluorescence microscope at 24 and 48 hpf. White arrowheads indicate DsRed-positive embryos.

```
Wt         TCAGCCTGTGACGACAGAGGAGGACCCC----------------------GAGCAGTGCTCCACATGCGAATTCA  53
#001/2566  TCAGCCTGTGACGACA----------------------------------GAGCAGTGCTCCACATGCGAATTCA  41(-12)
#002/2537  TCAGCCTGTGACGA----------------------------------CCGAGCAGTGCTCCACATGCGAATTCA  41(-12)
#003/2399  TCAGCCTGTGACGACAGTGCTCC---------------------------------------ACATGCGAATTCA  36(-17)
#004/1545  TCAGCCTGTGACGACAGAGGAGGACCCC----------------------GAGCAGTGCTCCACATGCGAATTCA  53(0)
#005/1424  TCAGCCTGTGACGACAGAGGAG---------------------------------GTGCTCCACATGCGAATTCA  42(-11)
#006/1362  TCAGCCTGTGACGACAGAGGAGG----------------------------AGCAGTGCTCCACATGCGAATTCA  47(-6)
#007/1339  TCAGCCTGTGACGAGCAGTGCTCC--------------------------------------ACATGCGAATTCA  37(-16)
#008/1291  TCAGCCTGTGACGACAGAGG----------------------------------AGTGCTCCACATGCGAATTCA  41(-12)
#009/1077  TCAGCCTGTGACGACAGAGGAG-------------------------------CAGTGCTCCACATGCGAATTCA  44(-9)
#010/ 469  TCAGCCTGTGCCGAGCAGTGCTCC--------------------------------------ACATGCGAATTCA  37(-16)
#011/ 378  TCAGCCTGTGACGACAGAGGAG-------------------------------GAGTGCTCCACATGCGAATTCA  44(-9)
#012/ 374  TCAGCCTGTGACGACAGAGGCTGAG--------------------------------GCTCCACATGCGAATTCA  43(-10)
#013/ 370  TCAGCCTGTGACGAC-----------------------------------GAGCAGTGCTCCACATGCGAATTCA  40(-13)
#014/ 326  TCAGCCTGTGACGACAGTGCGACA----------------------------------GACGACATGCGAATTCA  41(-12)
#015/ 322  TCAGCCTGTGACG------------------------------------CGAGCAGTGCTCCACATGCGAATTCA  39(-14)
#016/ 292  TCAGCCTGTGACGACAGTGCTGC---------------------------------------ACATGCGAATTCA  36(-17)
#017/ 283  TCAGCCTGTGACGACAGAGCTGTGC----------------------------------TCCACATGCGAATTCA  41(-12)
#018/ 276  TCAGCCTGTGACGACAGAGGAG--------------GCAGTGCAGTG------CAGTGCTCCACATGCGAATTCA  55(+2)
#019/ 262  TCAGCCTGTGACGACAGAGGAGT-----------------------------------GCAGGAGTGCGAATTCA  40(-13)
#020/ 259  TCAGCCTGTGACGACAGAGGAGGACC------------------------GAGCAGTGCTCCACATGCGAATTCA  51(-2)
#021/ 241  TCAGCCTGTGACGACAG-----------------------------TGACGAGCAGTGCTCCACATGCGAATTCA  46(-7)
#022/ 214  TCAGCCTGTGACGACAGAGGAGGTG-------------------------------TGCTCCACATGCGAATTCA  44(-9)
#023/ 206  TCAGCCTGTGACGACAGAGGAGG-----------------------------------ACC--AATGCGAATTCA  38(-15)
#024/ 199  TCAGCCTGTGACGACAGAGGAG-------GAGTGCAGTGCAC----G----AGCAGTGCTCCACATGCGAATTCA  60(+7)
#025/ 191  TCAGCCTGTGACGACAGAGGA----------------------------CGAGCAGTGCTCCACATGCGAATTCA  47(-6)
#026/ 186  TCAGCCTGTGACGACAGA-------------------------------CGAGCAGTGCTCCACATGCGAATTCA  44(-9)
#027/ 168  TCAGCCTGTGACGACAGAGGAG---------------------------------GAGCTCCACATGCGAATTCA  42(-11)
#028/ 165  TCAGCCTGTGACGACAGAGGAGGGC----------------------CCCGAGCAGTGCTCCACATGCGAATTCA  53(0)
#029/ 159  TCAGCCTGTGACGACAGAGGAG---------GTG---TGCTCCTCTG----AGCAGTGCTCCACATGCGAATTCA  59(+6)
#030/ 157  TCAGCCTGTGACGACAGAGGAGGA--------------------------GAGCAGTGCTCCACATGCGAATTCA  49(-4)
#031/ 154  TCAACCT--------------------------------------CAGCCGAGCAGTGCTCCACATGCGAATTCA  37(-16)
#032/ 142  TCAGCCTGTGACGACAG------------------------------------CAGTGCTCCACATGCGAATTCA  39(-14)
#033/ 134  TCAGCCTGTGACAACAGGTTGCC-------------------------CCGAGCAGTGCTCCACATGCGAATTCA  50(-3)
#034/ 132  TCAGCCTGTGACGACAG--------------------------------------GTGCTCCACATGCGAATTCA  37(-16)
#035/ 119  TCAGCCTGTGACGACTGTGCTCCACATGCT---------------CCACCGAGCAGTGCTCCACATGCGAATTCA  60(+7)
#036/ 107  TCAGCCTGTGACC-------------------------------------GAGCAGTGCTCCACATGCGAATTCA  38(-15)
#037/ 100  TCAGCCTGTGACGAG--------------------------------CCCGAGCAGTGCTCCACATGCGAATTCA  43(-10)
#038/  95  TCAGCCTGTGACGACAGCTGTGCTC--------------------CTGTCGAGCAGTGCTCCACATGCGAATTCA  55(+2)
#039/  94  TCAGCCTGTGACGACAGAGGAGGACCC-----------------------GAGCAGTGCTCCACATGCGAATTCA  52(-1)
#040/  90  TCAGCCTGTGACGACAGAGGAGG--------------------------CGAGCAGTGCTCCACATGCGAATTCA  49(-4)
#041/  83  TCAGCCTGTGACGACAGTGAGGA-----------------------CAGCGAGCAGTGCTCCACATGCGAATTCA  52(-1)
#042/  82  TCAGCCTGTGACGACA-------------------------------CTCGAGCAGTGCTCCACATGCGAATTCA  44(-9)
#043/  79  TCAGCCTGTGACGACACCACAC-------------------------ACCGAGCAGTGCTCCACATGCGAATTCA  50(-3)
#044/  77  TCAGC-------------------------------------------------AGTGCTCCACATGCGAATTCA  26(-27)
#045/  69  TCAGCCTGTGACGACAGAGGAGG---------------------------------------ACATGCGAATTCA  36(-17)
#046/  54  TCAGCCTGTGACGACAGGT-----------------------------CAGAGCAGTGCTCCACATGCGAATTCA  46(-7)
#047/  52  TCAGCCTGTGACGACAGAGGAGGA----------------------------GCTGTGCTCCACATGCGAATTCA  47(-6)
#048/  49  TCAGCCTGTGACGACAGAGGAGGGT----------------------------GTGTGCTCCACATGCGAATTCA  47(-6)
#049/  49  TCAGCCTGTGACGACA-------------------------------GTGACGCAGTGCTCCACATGCGAATTCA  44(-9)
#050/  41  TCAGCCTGTGACACCTCAGCCT-----------------------GCCCCGACCAGTGCTCCACATGCGAATTCA  52(-1)
#051/  40  TCAGCCTGTGACGACAGAGGAG------------------GTGCAGTGCCGAGCAGTGCTCCACATGCGAATTCA  57(+4)
#052/  40  TCAGCCTGTGACGACAGTGC---------------------------------------TCGTCATGCGAATTCA  36(-17)
#053/  39  TCAGCCTGTGACGACACCTCAG-------------------------------CAGTGCTCCACATGCGAATTCA  44(-9)
#054/  38  TCAGCCTGTGACGACAGAGGAGGG------TTAGGGTTAGGGTTAGTG-----CAGTGCTCCACATGCGAATTCA  64(+11)
#055/  37  TCAGCCTGTGACGACAGAGGAG-------------------------GCAGAGCAGTGCTCCACATGCGAATTCA  50(-3)
#056/  34  TCAGCCTGTGACGAC----------------------------------CGAGCGGTGCTCCACATGCGAATTCA  41(-12)
#057/  29  TCAGCCTGTGACGACAGAGGAG--------------------GCAGTG-----CAGTGCTCCACATGCGAATTCA  50(-3)
#058/  28  TCAGCCTGTGACGACAGA-----------------------------------------------TGCGAATTCA  28(-25)
#059/  24  TCAGCCTGTGACGACAGAGGAGGACCCC---------------------CGAGCAGTGCTCCACATGCGAATTCA  54(+1)
#060/  23  TCAGCCTGTGACGACAGAGGAGGTGCTG------------------CTCCGAGCAGTGCTCCACATGCGAATTCA  57(+4)
#061/  20  TCAGCCTGTGACGA-CAGAGGAGGACC---------------------CCGAGTAGTGCTCCACATGCGAATTCA  53(0)
#062/  19  TCAGCCTGT-------------------------------------TTGTTTGCAGTGCTCCACATGCGAATTCA  38(-15)
#063/  18  TCAGCCTGTGACGACAGAGGAG---------GAGTGTGACGACAATTTCCAAGCAGTGCTCCACATGCGAATTCA  66(+13)
#064/  18  TCAGCCTGTGACGACAGAGGAG-------TAGTGCAGTGCTCCACCG----AGCAGTGCTCCACATGCGAATTCA  64(+11)
#065/  18  TCAGCCTGTGACGACAGAGGAGT-----------------------------GCAGTGCTCCACATGCGAATTCA  46(-7)
#066/  17  TCAGCCTGTGACGACAGAGGAGGACC-------------------------AGCAGTGCTCCACATGCGAATTCA  50(-3)
#067/  16  TCAGCCTGTGACGACAGAACAG----------------------------------TGCTCCACATGCGAATTCA  41(-12)
#068/  16  TCAGCCTGTGACGACAGCAGTGGTC-------------------------GAGCAGTGCTCCACATGCGAATTCA  50(-3)
#069/  16  TCAGCCTGTGACGACAGTGCAGTG-----------------------CTCGAGCAGTGCTCCACATGCGAATTCA  52(-1)
#070/  15  TCAGCCTGTGACGACAG----------------------------------------GCTCCACATGCGAATTCA  35(-18)
#071/  13  TCAGCCTGTGACGACAGAGGAG-----------------GACAC-----------GTGCTCCACATGCGAATTCA  47(-6)
#072/  13  TCAGCCTGTGACGACAGTA------------------------------CGAGCAGTGCTCCACATGCGAATTCA  45(-8)
#073/  12  TCAGCCTGTGACGACAGAGGA--------------------------------------------TGCGAATTCA  31(-22)
#074/  12  TCAGCCTGTGACGACAGCACGA------------------------------GCAGTGCTCCACATGCGAATTCA  45(-8)
#075/  11  TCAGCCTGTGACGACAGAG----------------------------------CCGTGCTCCACATGCGAATTCA  41(-12)
#076/  11  TCAGCCTGTGACGACAGAGGAGC--------------------AGTGCAGGAGCAGTGCTCCACATGCGAATTCA  55(+2)
#077/  11  TCAGCCTGTGACGACAGTG-------------------------------GAGCAGTGCTCCACATGCGAATTCA  44(-9)
#078/  10  TCAGCCTGTGACGACAGAGGAG-----------------------------GGCAGTGCTCCACATGCGAATTCA  46(-7)
#079/   9  TCAGCCT--------------------------------------CAGCCGAGCAGTGCTCCACATGCGAATTCA  37(-16)
#080/   9  TCAGCCTGTGACAACA----------------------------------GAGCTGTGCTCCACATGCGAATTCA  41(-12)
#081/   9  TCAGCCTGTGACGACAG--------------------------------AGAGCAGTGCTCCACATGCGAATTCA  43(-10)
#082/   9  TCAGCCTGTGACGACAGAGGAGGACCC----------------------CGAGCAGTGCTTCACATGCGAATTCA  53(0)
#083/   8  TCAGCCTGTGACGACAGAG----------------------------------CGGTGCTCCACATGCGAATTCA  41(-12)
#084/   8  TCAGCCTGTGACGACAGAGGAGG-CCC-----------------------GAGCAGTGCTCCACATGCGAATTCA  51(-2)
#085/   8  TCAGCCTGTGACGAC---------------------------------------AGTACTCCACATGCGAATTCA  36(-17)
#086/   8  TCAGCCTGTGACGAC----------------------------------CGAGCCGTGCTCCACATGCGAATTCA  41(-12)
#087/   8  TCAGCCTGTGACGAGTAGTGCTCC--------------------------------------ACATGCGAATTCA  37(-16)
#088/   7  TCAGCCTGTGACAACA----------------------------------GAGCAGTGCTCCACATGCGAATTCA  41(-12)
#089/   7  TCAGCCTGTGACGACAGTGCAACA----------------------------------GACGACATGCGAATTCA  41(-12)
#090/   7  TCAGCCTGTGACGACTG------------------------------CCCGAGCAGTGCTCCACATGCGAATTCA  45(-8)
#091/   7  TCAGCCTGTGACGAGCAGTGA--------------------------------------TCCACATGCGAATTCA  37(-16)
#092/   6  TCAGCC------------------------------------------------AGTGCTCCACATGCGAATTCA  27(-26)
#093/   6  TCAGCCTGTGACGACAGAGGAGGAAGTGCTTAAGCAGTGGAGCAGTGCTTAAGCAGTGCTCCACATGCGAATTCA  75(+22)
#094/   6  TCAGCCTGTGACGACAGAGGAGGAGCA-----------------------GAGCAGTGCTCCACATGCGAATTCA  52(-1)
#095/   6  TCAGCCTGTGACGACAGAGGAG---------------------------------GTGCACCACATGCGAATTCA  42(-11)
#096/   6  TCAGCCTGTGACGACAGAGGAGTAG------------------------TGAGCAGTGCTCCACATGCGAATTCA  51(-2)
#097/   6  TCAGCCTGTGACGACAGAGG---------------------------------CAGTGCTCCACATGCGAATTCA  42(-11)
#098/   6  TCAGCCTGTGACGACAGAGGC-------------------------------GCAGTGCTCCACATGCGAATTCA  44(-9)
#099/   6  TCAGCCTGTGACGACCGAA----------------------------------CAGTGCTCCACATGCGAATTCA  41(-12)
#100/   6  TCAGCCTGTGACGACCG----------------------------------AGCAGTACTCCACATGCGAATTCA  41(-12)
#101/   5  TCAGCCTGTGACGACAGAAGCTGAG--------------------------------GCTCCACATGCGAATTCA  43(-10)
#102/   5  TCAGCCTGTGACGACAGAGGA---------------------------CCGAGCAGTGCTCCACATGCGAATTCA  48(-5)
#103/   5  TCAGCCTGTGACGACAGAGGAG---------GAGCAGTGCTCCG-------AGCAGTGCTCCACATGCGAATTCA  59(+6)
#104/   5  TCAGCCTGTGACGACAGAGGAG----TAGTGGAGTAGAGGAGTAGTG---GAGCAGTGCTCCACATGCGAATTCA  68(+15)
#105/   5  TCAGCCTGTGACGAC-----------------------------------GAGCAATGCTCCACATGCGAATTCA  40(-13)
#106/   5  TCAGCCTGTGCCGA--------------------------------------GCAGTGCTCCACATGCGAATTCA  39(-14)
```

Supplementary Figure S3.

Mutation sequences in TL2-injected embryos. One-cell eggs were injected with TL2-mRNA pairs (150 ng/μL each) into the yolk, and their genomic DNAs were extracted from ten embryos at 21 hpf. The DNA fragment including TL2-target sequence was amplified by PCR and subjected to amplicon deep-sequencing analysis. The sequence alignment was generated by ClustalW[1]. This is shown in order from the left: sequence IDs; read counts; sequences; sequence lengths; and the sizes of the insertions and deletions (-, deletions; +, insertions). The sequence IDs and the read counts coloured red indicate the frameshift mutation.

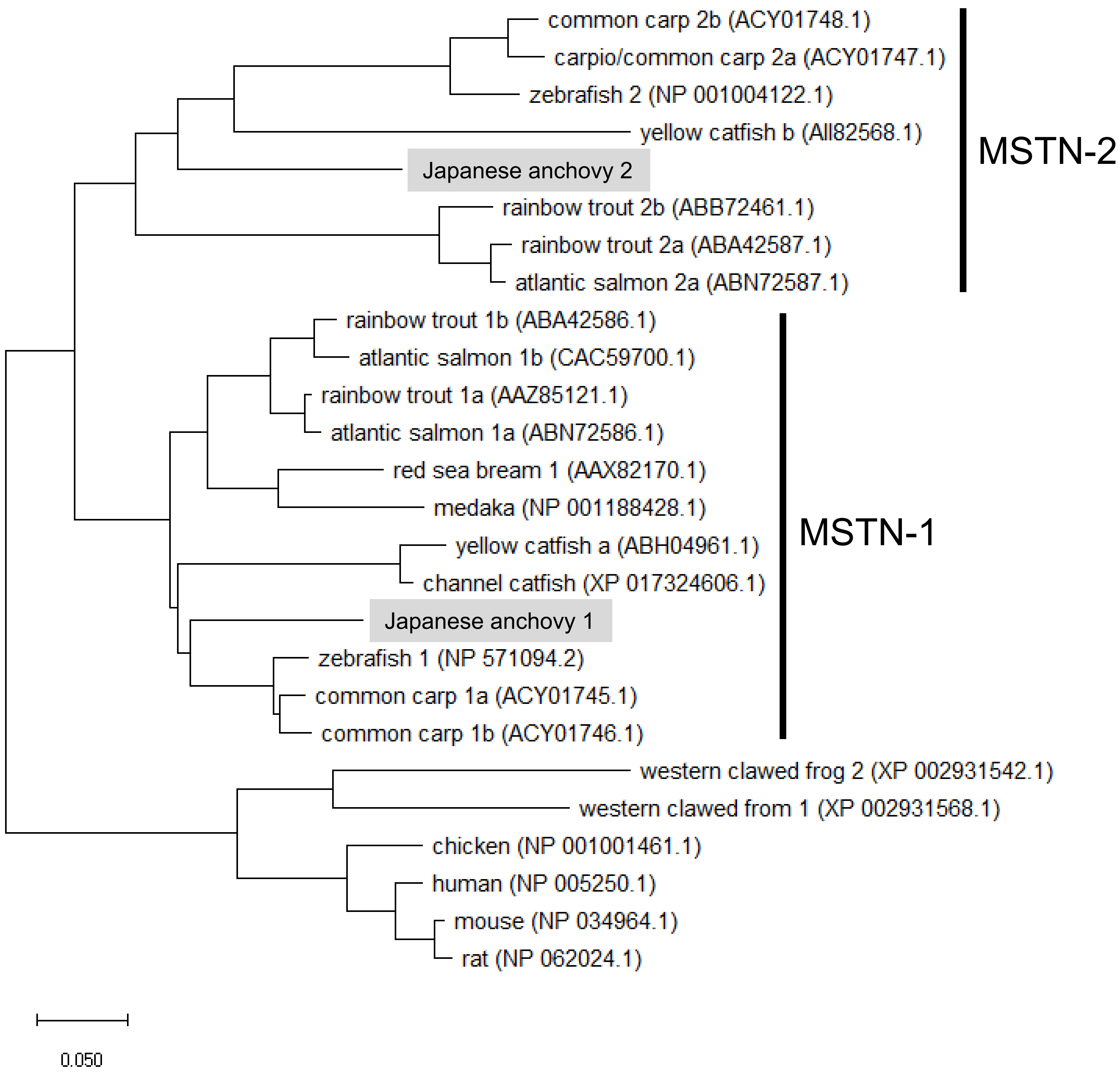

Supplementary Figure S4. Phylogenetic tree of teleost *MSTNs*.
Deduced amino acid sequences of MSTNs were analysed using the neighbour-joining method[2] with Molecular Evolutionary Genetics Analysis Software MEGAX[3]. The bar shows the number of amino acid substitutions per site. The accession numbers are shown in brackets. Japanese anchovy MSTN-1 and -2 are shaded in grey boxes.

Supplementary Reference

1. Larkin, M. A. *et al.* ClustalW and ClustalX version 2. *Bioinfomatics.* **23**, 2947-2948 (2007).

2. Saitou N. and Nei M. The neighbor-joining method: A new method for reconstructing phylogenetic trees. *Mol. Biol. Evol.* **4,** 406-425 (**1987**).

3. Kumar S., Stecher G., Li M., Knyaz C., and Tamura K. MEGA X: Molecular Evolutionary Genetics Analysis across computing platforms. *Mol. Biol. Evol.* **35**, 1547-1549 (**2018**).
